# Retrospective qualitative pilot study incorporating patients’ personal life aspects on admission to palliative care: What should we know about patients to give them the best possible care?

**DOI:** 10.1007/s00508-019-01552-5

**Published:** 2019-10-01

**Authors:** Anna Kitta, Feroniki Adamidis, Matthias Unseld, Herbert H. Watzke, Eva Katharina Masel

**Affiliations:** Clinical Division of Palliative Care, Department of Internal Medicine I, Medical University of Vienna, Waehringer Guertel 18–20, 1090 Vienna, Austria

**Keywords:** Respect, Personhood, Physician patient relationship, Communication, Medical history taking

## Abstract

**Background:**

This pilot study examined which of a patient’s personal aspects should be taken into account in a hospital setting on admission to the palliative care unit (PCU) by asking patients the question “what should I know about you as a person to help me take the best care of you that I can?”

**Methods:**

This retrospective study used qualitative methodology to thematically analyze answers from 14 patients admitted to the PCU of the Medical University of Vienna during July and August 2018. The question “what should I know about you as a person to help me take the best care of you that I can?” was asked on the day of admission, notes were taken during the interview and the patient’s answers were written out immediately afterwards. Data were analyzed using NVivo 12.

**Results:**

Results revealed four topics: characterization of one’s personality, important activities, social bonding, and present and future concerns regarding the patient’s illness. Data showed that this question enabled patients to describe themselves and what was important to them. This might result in an improved sense of self-esteem in patients and represents an opportunity for professionals to treat patients in a more individualized manner; however, patient reactions also revealed a reluctance to address certain personal issues within a medical context.

**Conclusion:**

The study results provide insights into the benefits of paying more attention to personal life aspects of severely ill patients on admission to a PCU. Addressing individual aspects of patients’ lives might improve the healthcare professional-patient relationship.

## Introduction

Taking a medical history mostly focuses on physical and health-related aspects of patients. Medical professionals ask about current symptoms, about former illnesses and record current medications, physical function, mental health, and family history, among other such information. The object of these questions is to generate a comprehensive picture of the individual as this is of essential importance for diagnostics and further procedures; however, these aspects might not allow a perception of the individual as a person rather than a patient, leaving out the “human” and only addressing the individual as a collection of signs and symptoms of their disease.

The Canadian psychiatrist Harvey Chochinov and his team developed dignity therapy to alleviate psychosocial distress and existential suffering of patients in palliative care settings. This therapy incorporates the patient’s biography into the care, in support of a sense of dignity and self-worth. It focuses on issues that are of importance to the individual patient and should be remembered [1, 2]. This takes several days and results in a written text that is then provided to the patient and caregivers. Such a practice raises the question of whether it is feasible to provide patients the time to talk about who they are in a busy hospital setting. In consideration of the limited time and personnel resources in hospitals, the following study investigated what effects the question “what should I know about you as a person to help me take the best care of you that I can?” had on patients admitted to a palliative care unit (PCU), and was asked during the taking of a medical history. The question relates to Chochinov’s previous studies where it was mentioned as an example [3, 4]. This qualitative study retrospectively analyzed reactions and answers from patients to this question on the day of admission to the PCU. The PCU of the Medical University in Vienna is a hospital-based unit consisting of 12 beds with approximately 260 patients admitted each year. The average stay compromises 1–3 weeks and approximately 40% of the patients can be discharged after the stay in the unit.

## Patients, materials and methods

### Participants and data collection

Over a period of several months starting in July 2018, patients admitted to the PCU of the Medical University of Vienna were asked on the day of admission “what should I know about you as a person to help me take the best care of you that I can?” by a palliative care physician in training (AK) after taking the patient’s medical history. Handwritten notes were taken while listening to the patients, and a full transcription followed in the form of a memorandum immediately after the conversation. This memorandum also included surrounding conditions while the conversation took place. After realizing the diversity of the answers, in Fall 2018 it was agreed to retrospectively analyze the documentation. The study was approved by the medical ethics committee of the Medical University of Vienna (approval no. 2003/2018). Subsequently the documentation of the first 2 months was analyzed, which were for July and August. The inclusion criteria for patient data were the following: the patient was over 18 years of age, provided no reasons for nonparticipation such as cognitive deficits, delirium, mental illness, excessively poor performance status or severe septicemia with impaired consciousness and no language problems.

### Data analysis

Statements in German were translated into English by a professional translator and in this case transcripts were not returned to the participants for comments or corrections. Data were interpreted using a qualitative thematic analysis. A set of codes was generated via a careful re-reading of the answers. The codes provided structures of order and indicators of relevant answer characteristics. Systemization, comparison, and the generation of themes followed. The codes were subsequently assigned to different themes to correlate statements, make connections, and elucidate differences among answers to understand the meanings underlying the statements as well as the context and circumstances of the conversations [5, 6]. The software program NVivo 12 (QSR International) was used to analyze the data. An overview of codes and topics is provided in Table 1.

**Table 1:** Overview of the analysis structure used

Topic	Themes	Categories
Worth knowing for best possible care	Characterization of the personality	Preferences
		Positive characteristics
		Sensitive features
		External view
	Important activities	Work
		Hobbies
	Social bonding	Family
		Friends
		Current care
		Connections to palliative care team
	Present and future concerns regarding the patients’ illness	Background
		Medical history
		Body and symptoms
		Psyche
		Wishes/hopes
		Dying
	Reactions and emotions	Reluctance
		Crying
		Laughing

## Results

A total of 14 patients met the inclusion criteria, including 5 women (36%) and 9 men (64%). The mean age was 60 years (range 45–89 years) with a mean Karnofsky index of 52% (range 20–70%), indicating that the individuals needed extensive support and frequent medical attention. The Karnofsky performance status scale (KPS) was used to rate patient functional status. The KPS ranges from 0 to 100, with 0 signifying death and 100 indicating perfect health [7]. All respondents suffered from advanced cancer, most commonly breast cancer (*n* = 3, 21%) and pancreatic cancer (*n* = 3, 21%), followed by colon cancer (*n* = 2, 14%). Other diseases included tonsillar cancer, leiomyosarcoma, lung cancer, ovarian cancer, and gastric neuroendocrine tumor at *n* = 1 (7%) each. All respondents were interviewed on the day of admission or transfer to the PCU. For four patients, relatives were present during the interview, two patients had already been admitted one time prior to the current admission and all other patients were admitted to the PCU for the first time. An overview of all participants is provided in Table 2.

**Table 2:** Profile of the study participants

ID	Cancer disease	Age (years)	ECOG (0–4)	Karnofsky index (%)	m/f
*P01*	Breast cancer	45	4	20	f
*P02*	Tonsillar cancer	50	3	50	m
*P03*	Leiomyosarcoma	79	3	50	f
*P04*	Lung cancer	68	3	50	m
*P05*	Colorectal cancer	81	2	60	m
*P06*	Ovarian cancer	90	3	50	f
*P07*	Pancreatic cancer	89	2	60	m
*P08*	Breast cancer	53	2	50	f
*P09*	Gastric neuroendocrine tumor	74	2	60	m
*P10*	Pancreatic cancer	75	2	60	m
*P11*	Meningioma	49	1	70	m
*P12*	Breast cancer	81	4	50	f
*P13*	Colorectal cancer	47	2	60	m
*P14*	Pancreatic cancer	55	4	40	m

*ECOG* Eastern Cooperative Oncology Group, *ID* identification, *m* male, *f* female

An analysis of the transcripts revealed the following four themes that are presented as a narrative using patient quotations to explore each theme in depth: characterization of the personality, important activities, social bonding, and present and future concerns regarding the patient’s illness. The interviews were marked with P x (patient number “x”) or P + x (caregiver of patient number “x”) and are presented in detail later.

### Qualitative findings

#### Initial reaction

A significant portion of the patients appeared irritated when asked the question “what should I know about you as a person to help me take the best care of you that I can?” Approximately half of respondents answered right away but the other half hesitated. Of the patients two reacted nonverbally with physical gestures, a shrug of the shoulders and simultaneous shaking of the head, followed by a negative or non-answer (P02, P04), two other patients listened to the question until the end, then nodded without any further reaction. For example, patient 10 replied, “you can ask anything.” (P10) while patient 11 responded, “please” (P11). In these cases, the question was repeated. These patients did not seem to understand it as a question at first, but more as an introduction to personal questions that would subsequently be asked. In four cases other persons responded with a counterquestion. The husband of patient 1 asked, “from a medical point of view?” (P + 01) while patient 12 asked, “as a human being?” (P12). After the clarification, “generally speaking about you as a person”, both patients were able to answer. Patient 7 replied, “yes, what should I say?” (P07) but continued to speak afterwards. Patient 13 also replied, “yes, what should I say?” (P13), whereupon the roommate interrupted, saying he could talk about his hobbies or preferences. The patient then responded in kind. In two cases (P + 01, P + 04), the question was partly answered by relatives, as patients and their family members or loved ones were free to decide whether to be present during the conversation. The relatives responded with answers to the questions before the patients themselves, and as such, these relatives’ answers were also included in the analysis. During the conversation with two patients (P06, P14), the question led to a long break in the conversation. This silence was not interrupted, and respondents began to speak after a period of time.

#### Theme 1: characterization of one’s own personality

Respondents initially discussed personal information focused on their basic needs, such as food (“I eat everything”, P09) and sleeping (“I need silence for sleeping”, P05 and “I sleep poorly. I am active at night”, P13).

Patients also provided adjectives about themselves in an apparently honest manner; these included generally positive attributes as well as sensitive characteristics. Descriptions such as “happy, positive person” (P + 01), “a lot of compassion” (P06), “easy” (P09) and “good person” (P12) were mentioned. Patient 10 said that he had been “very active” and “travelled a lot” (P10) and patient 6 stated that she was “socially very involved” (P06); however, people also provided more sensitive information, such as “yes, I can really jangle everyone’s nerves” (P13). Patient 5 indicated a need for rest and calm. The wife of patient 4 added after his answer “his anxiety disorder is already known, that is not due to the disease now. He has always been scared” (P + 04).

Feedback from those accompanying the patient to the PCU often led to the divulging of additional information either by relatives who supplemented patient answers or from the patients themselves. The partner of patient 1 added, “she was always there for us, encouraged us and […] she was a kindergarten teacher! All children loved her!” (P + 01). Patient 6 used the narration of an episode from her youth regarding feedback from her examiners in the context of her training as a nurse to add personal details, “after 6 weeks of school we always had to work for 2 months half a day on the wards. Afterwards, there was to be an assessment. But with my complexes, I feared an annihilating judgement, I had diarrhea, but there was no getting out and then the judgment was: ‘the sunshine of the department’!” [patient started to cry] (P06); however, patient 8 responded by saying, “you know much better what I need.” (P08).

#### Theme 2: important activities

The patients also talked about professions and leisure activities, providing additional perspectives. As mentioned, patient 1 was a kindergarten teacher. Patient 9 reported that he had worked as a truck driver, and patient 13 explained his night time activity by mentioning former jobs as a warehouse worker, deliveryman and supplier of a bakery, and night waiter at a bar. Patient 6, who provided a long, fluid, and coherent account of her origins, life path, and current situation, stated that her job as a nurse was central to her life. “I have been a widow for 40 years and have done my job with love—I would do it again if I was born again” (P06). She came back to this several times, as in a report on her youth: “I could only learn a profession that didn’t cost anything. I did not know then that it was the profession [for me]. But it was” (P06).

Hobbies were also reported, such as working in the garden (P07), crafts, fishing and reading fishing magazines (P09), traveling and walking (P10), and reading and going to the opera, theater, and concerts (P06). These personal aspects would have otherwise been unknown to the hospital team.

#### Theme 3: social bonding

Participants frequently mentioned their family and friends in describing their own characteristics. Patient 10 stated, “yes I am a family person. I have two grown children, four grandchildren.” (P10), while patient 6 reported having a “loving son, who cares enormously, almost too much for his life. Additionally, I have good friends who visit me” (P06) and went on to mention her seven siblings, and that she was a “daddy’s girl” but also appreciated her mother a great deal. She also added how she is currently cared for in the nursing home and mentioned an appreciation for her social network there. Patient 12 started to speak, “My daughter …” (P12), then started to cry and said nothing else. Of the patients two referred to existing social bonds with their treatment team. Patient 7 emphasized his daughter-in-law, who worked in the hospital and was well-known in the unit, while patient 3 responded, “during the last visit I did an advance directive with Prof. (senior physician on the ward)” (P03) and indicated that the patient was already known on the unit.

#### Theme 4: present and future concerns regarding the patient’s illness

Patients mentioned physical and psychosocial complaints several times, included as items relating to symptom burden. Common symptoms mentioned were pain (P07, P06, P10), fatigue and weight loss (P09), and constipation (P06). Patients also brought up issues that were not obvious at first glance. Patients 6 and 9 reported increasingly poor eyesight. Patients 6 and 14 spoke of reduced hearing and its consequences, “I often do not know if I express and say everything correctly. It is a dilemma. And then it may be that you talk at cross purposes” (P14). Patient 6 provided information on her inner experience, “so far, I come to terms with the disease well. Only a few weeks, maybe 2 months ago, the deterioration started. Maybe longer […] and so it slowly went downhill. […] It’s a fight” (P06). Patient 9 said, “being tired all the time bothers me […]. Aside from that I cannot believe that I am ill—I feel nothing.” (P09). Wishes concerning their future stay in the PCU as well as thoughts concerning the imminence of death were also expressed during several interviews. Patient 7 stated, “I have no special wishes. Yes, except the pain that should be under control. And otherwise—yes I have a garden and I think you have to use it and that’s why I do not want to stay here for too long” (P07). Patient 6 more specifically addressed approaching death, “It’s a nice feeling to be safe and I wish I could die fast and—as far as I can—(have the possibility to) say goodbye. I do not want life-prolonging measures, but, if possible, a relief” (P06). Patient 9 also mentioned the topic of dying, “sometimes I hope, hopefully I’ll be dead soon.” (P09), but in the next sentence turned to a discussion of his hobbies and physical complaints. Interviewees also shared ambivalence and occasionally provided in-depth answers. Some patients, however, did not answer or hardly answered the question (P02, P04) at all.

## Discussion

This pilot study explored whether a personal question might add personal depth to the patient’s medical history in addition to the list of physical symptoms and assessments of deficits and functions. The results of this study suggest that patient responses to the question, “what should I know about you as a person to help me take the best care of you that I can?” differed in tone and content from other questions asked when taking a medical history. Besides physical symptoms, personal aspects and emotions were discussed, and in some cases revealed the person behind the illness. Answers ranged from in-depth, chronological life stories to social, intimate, and emotional life details, and in some, a reluctance to answer the question. The responses also demonstrated that relatives were motivated to respond to questions as well. This could be due to a desire to support the treatment team in learning as much as possible about the individual patient to be able to provide better care.

In this study two patients had already been admitted some time prior to the current admission, all other patients were admitted to the PCU for the first time. In the case of a repeated admission, we would not recommend asking the same question once again but rather reuse some of the personal information noted in the past to create a welcoming atmosphere. While the questions sometimes yielded intimate insights into the patients’ lives, other patients were irritated by the question. For them, answering it seemed more challenging than responding to the more familiar, illness-oriented questions asked by medical practitioners in a hospital setting.

Chochinov stated that how patients experience being perceived by others is of great importance for their dignity and self-worth [3]. He observed that patients conveyed some aspects of their life stories as though they knew this information could improve their care [4, 8]. As Breitbart mentioned from the point of view of the palliative care team, “for our patients we are […] focused on preserving the ‘who’ of the patient, preserving the someone who that patient has been in life, and allowing that to be what is most meaningful and significant as they face death” [9].

Palliative care aims to provide comprehensive end of life care. By definition, this includes physical, psychosocial, and spiritual dimensions [10–12]. The total pain concept can be understood as a perspective that covers not only physical suffering but the social and mental aspects of illness as well, especially regarding the struggle to find meaning in a life that has changed profoundly through the experience of severe disease [13].

Several assessment tools in palliative care exist that concentrate on different symptoms, such as Spict, Pers(2)on, and IPOS [14–16]. Professional healthcare givers, who additionally value the individual patient by expressing an interest in who he or she is as a person, strengthen the patient’s sense of dignity. Based on this finding, Chochinov developed the dignity therapy that corresponds to a type of short-term psychotherapy for critically ill people with limited time remaining to alleviate psychosocial and existential distress. The intervention consists of discussing issues that are essential and meaningful for the individual, and that should be remembered via a conversation between interviewer and patient. The interview results in the development of an edited transcript of the interview to produce a document that is presented to the patient and can also be provided to caregivers. Chochinov’s approach can be helpful when attempting to support the integrity and dignity of people living with severe illnesses. It enables patients to reveal important aspects of their lives and show their individuality [17]. The process of dignity therapy takes several days and requires trained practitioners to take the time to listen, write, and edit the patient’s responses, and develop the final manuscript. In the same regard, Breitbart et al. developed individual meaning-centered psychotherapy for the treatment of psychological and existential distress that was found to yield significant benefits [18], emphasizing the fact that, “[i]t is a matter of being treated with respect, as a human being worthy of respect, worthy of being valued; valued by oneself and by the world one interacts with” [19].

Due to resource and time constraints, these therapies can be challenging for hospital PCUs; however, the goal of maintaining dignity is important to these institutions. In summary, we contend that asking, “what should I know about you as a person to help me take the best care of you that I can?” can be implemented into the time and resources expended by personnel taking patient histories.

### Limitations

Limitations of this study include that it was performed at a single, hospital-based unit, and the study results thus may not be generalizable. Findings also revealed that some patients were irritated or needed additional help to answer the question. Patients might not be used to being asked such a personal question in the context of a hospital stay and might not be aware of what is meant by the question or how they should respond. Answers rarely concerned political or spiritual beliefs but physical information and symptoms were often discussed. Answers also might differ depending on whether the question was asked at the beginning or end of the medical history. It would be interesting to examine if more concrete and less complex questions such as “what is important to you in life?” or the invitation “tell me about yourself” might be easier to respond to or if a question phrased as follows, “I will be your doctor, and so I have to learn a great deal about your body and your health and your life. Please tell me what you think I should know about your situation” [20] would result in less hesitation.

What is novel and relevant in this study is that we were able to provide direct information regarding how patients in a PCU react when being asked a personal question on admission. Further investigations examining how this personal information can be used by the treatment team, whether discussed in team meetings or added as notes in the patient’s chart, are recommended. It would also be interesting to study what effects access to this personal information has on team members and their relationships with patients.

## Conclusion

The results of the current study demonstrate that the question, “what should I know about you as a person to help me take the best care of you that I can?” signals personal interest in patients and can yield valuable information in a hospital setting for healthcare professionals [21]. In this study, relevant additional information about the patients was disclosed and a few intimate moments occurred. We also hypothesize that asking such a question might generate positive feelings in patients as they experience being perceived as individuals. Asking this type of question about personal topics may not only benefit patients but also enable professional healthcare givers to provide more individualized care.
